# The Genome Sequence of *Alcaligenes faecalis* NBIB-017 Contains Genes with Potentially High Activities against *Erwinia carotovora*

**DOI:** 10.1128/genomeA.00222-16

**Published:** 2016-04-07

**Authors:** Xiaoyan Liu, Daye Huang, Jinping Wu, Cui Yu, Ronghua Zhou, Cuijun Liu, Wei Zhang, Jingwu Yao, Meng Cheng, Suxia Guo

**Affiliations:** a National Biopesticide Engineering Technology Research Center, Hubei Biopesticide Engineering Research Center, Hubei Academy of Agricultural Sciences, Wuhan, China; b The Economic Crop Research Institute, Hubei Academy of Agricultural Sciences, Wuhan, China; c Department of Horticulture, Hubei Vocational College of Bio-technology, Wuhan, China; d Beijing Jin Se Nong Hua Seeds Technology Inc., Beijing, China; e Haikou Experimental Station, Chinese Academy of Tropical Agricultural Sciences, Haikou, China

## Abstract

*Alcaligenes faecalis* NBIB-017, a Gram-negative bacterium, was isolated from soil in China. Here, we provide the complete genome sequence of this bacterium, which possesses a high number of genes encoding antibacterial factors, including proteins and small molecular peptides.

## GENOME ANNOUNCEMENT

*Alcaligenes faecalis* is a Gram-negative bacterium found in soil and water. Some *A. faecalis* spp. are capable of degrading pesticides such as endosulfan (1, 2) and 2,4-dichlorophenoxyacetic acid (3), as well as other inorganic compounds found in wastewater (4–6), while other strains of this species can produce nonnatural amino acids (7). However, *A. faecalis* strains are widely used in today’s agriculture and pharmaceutical industries (8).

Strain NBIB-017 was isolated from a rice paddy field in Hubei Province, China, in 2013. Its 16S rRNA sequence is 99.0% similar to that of *A. faecalis* NBIB-017. Whole-genome sequencing of *A. faecalis* NBIB-017 was performed using a strategy involving Solexa paired-end sequencing technology. Two libraries containing 400-bp and 750-bp inserts were constructed. Sequencing was performed with the paired-end strategy of 500-bp reads to produce 295 Mb and 320 Mb of ﬁltered sequences, representing 147-fold coverage, with an Illumina Solexa IIx genome analyzer (Wuhan Yanxing Biotechnology Co., Ltd., Wuhan, China). The reads were assembled into 20 contigs and 21 scaffolds using the SOAP *de novo* alignment tool (http://soap.genomics.org.cn/index.html#intro2). The gaps within and between the scaffolds were ﬁlled by sequencing the relevant PCR products by primer walking, with the use of an ABI 3730 capillary sequencer.

The NBIB-017 genome consists of a 4,190,208-bp chromosome with a G+C content of 56.46%. The chromosome consists of 3,900 coding sequences, 5 rRNA operons, and 52 tRNAs. Genome annotation was performed using the NCBI Prokaryotic Genome Automatic Annotation Pipeline, GenBank (nonredundant), Kyoto Encyclopedia of Genes and Genomes (9), and Clusters of Orthologous Groups (10) databases for BLASTp identiﬁcation (11). Four gene clusters in NBIB-017, covering 2.2% of its whole genome, are involved in antibiotic synthesis, namely, streptomycin, ansamycin, vancomycin, and novobiocin. Several degradation pathways were detected, including those involving benzoate, DDT, bisphenol, fluorobenzoate, dioxin, and xylene.

### Nucleotide sequence accession numbers.

The complete sequence of *A. faecalis* NBIB-017 has been deposited in NCBI under the accession number LNOL00000000. The strain is available from the China Center for Type Culture Collection (Wuhan, China) under the accession number CCTCC M 2015089.

## References

[1] Joo HS, Hirai M, Shoda M 2005 Characteristics of ammonium removal by heterotrophic nitrification-aerobic denitrification by *Alcaligenes faecalis* no. 4. J Biosci Bioeng 100:184–191. doi:10.1263/jbb.100.184. 16198262

[2] Kong L, Zhu S, Zhu L, Xie H, Wei K, Yan T, Wang J, Wang J, Wang F, Sun F 2014 Colonization of *Alcaligenes faecalis* strain JBW4 in natural soils and its detoxification of endosulfan. Appl Microbiol Biotechnol 98:1407–1416. doi:10.1007/s00253-013-5033-4. 23812277

[3] Shoda M, Ishikawa Y 2014 Heterotrophic nitrification and aerobic denitrification of high-strength ammonium in anaerobically digested sludge by *Alcaligenes faecalis* strain no. 4. J Biosci Bioeng 117:737–741. doi:10.1016/j.jbiosc.2013.11.018. 24411668

[4] Jiang L 2013 Effect of nitrogen source on curdlan production by *Alcaligenes faecalis* ATCC 31749. Int J Biol Macromol 52:218–220. doi:10.1016/j.ijbiomac.2012.10.010. 23085490

[5] Xia Z 2013 Effect of Tween 80 on the production of curdlan by *Alcaligenes faecalis* ATCC 31749. Carbohydr Polym 98:178–180. doi:10.1016/j.carbpol.2013.05.073. 23987333

[6] Honda N, Hirai M, Ano T, Shoda M 1998 Antifungal effect of a heterotrophic nitrifier *Alcaligenes faecalis*. Biotechnol Lett 20:703–705. doi:10.1023/A:1005382810088.

[7] Quiroz-Castañeda RE, Mendoza-Mejía A, Obregón-Barboza V, Martínez-Ocampo F, Hernández-Mendoza A, Martínez-Garduño F, Guillén-Solís G, Sánchez-Rodríguez F, Peña-Chora G, Ortíz-Hernández L, Gaytán-Colín P, Dantán-González E 2015 Identification of a new *Alcaligenes faecalis* strain MOR02 and assessment of its toxicity and pathogenicity to insects. Biomed Res Int 2015:570243. doi:10.1155/2015/570243. 25667924 PMC4312618

[8] Nakano M, Niwa M, Nishimura N 2014 Development of a PCR-based method for monitoring the status of *Alcaligenes* species in the agricultural environment. Biocontrol Sci 19:23–31. doi:10.4265/bio.19.23. 24670615

[9] Kanehisa M, Araki M, Goto S, Hattori M, Hirakawa M, Itoh M, Katayama T, Kawashima S, Okuda S, Tokimatsu T, Yamanishi Y 2008 KEGG for linking genomes to life and the environment. Nucleic Acids Res 36:D480–D484. doi:10.1093/nar/gkm882. 18077471 PMC2238879

[10] Tatusov RL, Galperin MY, Natale DA, Koonin EV 2000 The COG database: a tool for genome-scale analysis of protein functions and evolution. Nucleic Acids Res 28:33–36. doi:10.1093/nar/28.1.33. 10592175 PMC102395

[11] Tatusova TA, Madden TL 1999 BLAST 2 sequences, a new tool for comparing protein and nucleotide sequences. FEMS Microbiol Lett 174:247–250. doi:10.1111/j.1574-6968.1999.tb13575.x. 10339815

